# Cross-infection by multiple soil-borne pathogens alters the physicochemical properties and microbial community structure of tobacco rhizosphere soil

**DOI:** 10.1186/s12866-026-05173-7

**Published:** 2026-05-21

**Authors:** Yanan Ruan, Chi Wang, Xiaodong Shao, Zuoxin Tang, Tiyuan Xia, Zebin Chen

**Affiliations:** 1 College of Agricultural and Life Sciences, Kunming University, Kunming, 650214 China; 2 State Key Laboratory for Conservation and Utilization of Bio-Resources in Yunnan, Yunnan Agricultural University, Kunming, 650201 China; 3 Agricultural Environment and Resources Institute, Yunnan Academy of Agricultural Sciences, Kunming, Yunnan 650201 China; 4 Honghe Branch of Yunnan Tobacco Company, Mile, Yunnan 652300 China

**Keywords:** Healthy tobacco, Diseased tobacco, Physicochemical properties, Enzyme activity characteristics, Rhizosphere soil microorganisms

## Abstract

Soil-borne diseases of tobacco roots and stems, caused by pathogens including *Ralstonia solanacearum* (bacterial wilt), *Phytophthora parasitica* var. *nicotianae* (black shank), and root-knot nematodes (*Meloidogyne* spp.), represent critical threats to tobacco cultivation. However, information regarding alterations in rhizosphere soil properties and microbial communities following co-infection by multiple soil-borne pathogens remains limited for tobacco-growing regions in Luxi County, Honghe Prefecture, Yunnan Province, China. This study therefore investigated rhizosphere soils collected from healthy and diseased tobacco plants in Baishui Town. Eighteen soil physicochemical parameters and six enzyme activities were measured. Bacterial and fungal community compositions were analyzed using high-throughput sequencing targeting the 16S rDNA V4 region and the ITS1 region, respectively. Relationships between environmental factors and microbial community shifts were further explored using redundancy analysis (RDA) and Spearman correlation analysis. Results demonstrated that, compared with healthy tobacco plants, various physicochemical factors and enzyme activities in the rhizosphere soils of diseased plants were significantly elevated. Bacterial community diversity and species richness in diseased tobacco rhizosphere soils were significantly altered by pathogen infection. Specifically, the relative abundance of the phylum Proteobacteria significantly decreased by 18.20%, whereas Chloroflexi and Gemmatimonadetes significantly increased by more than two-fold. The fungal phylum Chytridiomycota significantly decreased by 72.68%. At the genus level, the beneficial bacterium *Sphingomonas* significantly decreased by 36.13%, and the beneficial fungus *Penicillium* significantly decreased by 90.59% in relative abundance. Conversely, pathogenic fungi *Fusarium*, *Phoma*, and *Plectosphaerella* significantly increased by 3-fold, 5-fold, and 3-fold, respectively.

RDA and Spearman correlation analyses revealed that variations in rhizosphere microbial communities were significantly and positively correlated with elevated soil pH, organic matter, total nitrogen, available nitrogen, total phosphorus, manganese content, and the activities of peroxidase and protease, while significantly and negatively correlated with decreased total potassium, available potassium, and boron contents. Our findings indicate that the imbalance (or alternatively: *dysbiosis of microbial communities coupled with the disruption*) of soil physicochemical properties, enzyme activities, and microbial community structure constitutes a typical characteristic of tobacco rhizosphere soils subjected to multiple pathogen co-infections in this region.

**Supplementary Information:**

The online version contains supplementary material available at 10.1186/s12866-026-05173-7.

## Introduction

The rhizosphere is a narrow zone of soil surrounding plant roots, where physical, chemical, and biological properties differ distinctly from those of bulk soil due to root activities [1]. Within this microenvironment, specific microecological conditions are established through interactions among plants, soil, and microorganisms [2]. Among these components, microorganisms represent the key drivers of the soil microecosystem, playing crucial roles in nutrient cycling, maintaining and improving soil fertility, suppressing soil-borne pathogens, enhancing plant stress resistance, and regulating host immunity [3–5]. Soil-borne pathogens can survive in soil for extended periods and infect plants by colonizing root xylem tissues under favorable conditions, rendering disease management challenging. However, certain soils possess natural suppressive ability against plant diseases even when pathogens are present; this ability is attributed, at least in part, to the microbial communities associated with plant roots [6,7]. Numerous studies have shown that specific microbial communities assist plants in resisting pathogen invasion through mechanisms such as niche competition, antibiotic production, pathogen suppression, and induction of plant systemic resistance [4,8–10]. Consequently, modulating microbial communities is considered a promising strategy for improving soil health and achieving sustainable disease control.

Soil-borne diseases are commonly accompanied by imbalances in the rhizosphere microecosystem, manifesting as multidimensional responses characterized by disrupted microbial community structure, altered soil physicochemical properties, and abnormal enzyme activities. At the microbial level, diseased plant rhizospheres generally exhibit an antagonistic shift characterized by the decline of beneficial microbiota (e.g., *Bacillus*, *Agromyces*, *Micromonospora*, and *Pseudonocardia*) and the enrichment of pathogens [10, 11]. However, distinct crops exhibit differential responses to pathogen invasion: Verticillium wilt in cotton leads to fungal community expansion and bacterial community contraction [12], bacterial wilt in tomato primarily reduces bacterial community diversity [13], and continuous cropping stress in ginseng is accompanied by concurrent decreases in both bacterial and fungal alpha diversity [14]. At the soil functional level, pathogen invasion typically induces significant alterations in pH, organic matter, and readily available nutrients (available nitrogen, available potassium, and available phosphorus) [14–16], concomitant with imbalances in key enzyme activities such as catalase, invertase, and urease [11,17]. These variations in physicochemical factors are recognized as key drivers reshaping microbial community structure. Although these studies have elucidated response patterns of rhizosphere microecology under single-disease conditions, research regarding the synergistic stress responses of the rhizosphere microecosystem to multiple soil-borne disease co-infections remains insufficient. Yunnan Province represents a major tobacco-producing region in China, generating approximately 750,000 tons of tobacco leaves annually and accounting for 50% of the national output [18]. Within this province, the Honghe tobacco-growing region suffers from year-round combined infestations of bacterial wilt, black shank, and root-knot nematode diseases. Co-infection by multiple pathogens frequently occurs in affected areas, significantly complicating disease management. Despite the well-documented critical role of microbial community diversity in resisting pathogen invasion [5, 10, 16, 19], research on the rhizosphere microecological characteristics of healthy and diseased tobacco in Luxi County, Honghe Prefecture, has not been conducted. Therefore, investigating the relationships among microbial communities, physicochemical factors, and enzyme activities in the tobacco rhizosphere under combined disease threats is of significant importance for understanding the microecological implications of multi-pathogen co-infection.

Accordingly, this study examined a tobacco field in Baishui Town, Luxi County, Honghe Prefecture, that had been continuously cultivated with tobacco for over five years, to collect rhizosphere soil samples from healthy tobacco plants and those co-infected with bacterial wilt, root-knot nematode, and black shank diseases. Through high-throughput sequencing of 16S rDNA V4 and ITS1 regions combined with analyses of soil physicochemical properties and enzyme activities, we addressed the following questions: (1) What are the variation characteristics of microbial community structure and diversity in the tobacco rhizosphere under combined disease infection? (2) What are the relationships between rhizosphere microecological alterations and soil physicochemical factors and enzyme activities? The findings will provide fundamental data for characterizing the microbial ecology of soil-borne tobacco diseases in this region, offering baseline support for subsequent in-depth investigations.

## Materials and methods

### Sample collection

Soil samples were collected in July 2018 from a field under continuous flue-cured tobacco cultivation for over five years in Baishui Town, Luxi County, Honghe Hani and Yi Autonomous Prefecture, Yunnan Province, China (103°30′00″ E, 24°46′00″ N). The cultivar K326 was used. Within the same field block characterized by uniform natural conditions, soil type, and agronomic management, two treatment groups were established based on plant phenotypes: the diseased group (FB), exhibiting typical soil-borne disease symptoms including stunted growth, leaf yellowing, and wilting; and the healthy group (CK), comprising asymptomatic plants with normal vigor. Six plants were selected per group and randomly allocated into three subgroups of two plants each.

For rhizosphere soil collection, the surface soil layer (2–3 cm) was removed before the plants were carefully uprooted. After shaking off loosely adhering bulk soil, the soil tightly adhering to root surfaces was collected using sterile soft brushes and designated as rhizosphere soil. Rhizosphere soils from the two plants within each subgroup were pooled and thoroughly homogenized to form one composite sample, yielding three biological replicates per group (*n* = 3). This pooling strategy was necessitated by the limited quantity of rhizosphere soil obtainable from individual plants (~ 15–20 g per plant), which was insufficient for the comprehensive suite of subsequent analyses. These composite samples were partitioned for (1) analyses of soil physicochemical properties and enzyme activities and (2) storage at − 80 °C for subsequent microbial DNA extraction.

### Soil physicochemical properties analyses

Soil physicochemical properties were determined according to standard methods described by Bao [20]. Soil pH was measured potentiometrically in deionized water at a soil-to-water ratio of 1:2.5 (w/v). Soil organic matter (SOM) was quantified by dichromate oxidation. Total nitrogen (TN) was determined by the Kjeldahl method, total phosphorus (TP) by molybdenum blue colorimetry following alkali fusion, and total potassium (TK) by flame photometry. Available nitrogen (AvN) was extracted by alkaline hydrolysis diffusion, available phosphorus (AvP) by molybdenum blue colorimetry, and available potassium (AvK) by flame photometry. Exchangeable calcium (ExCa) and magnesium (ExMg) were measured by atomic absorption spectroscopy (AAS) or inductively coupled plasma optical emission spectrometry (ICP-OES). Available micronutrients (Cu, Zn, Fe, Mn, B, S, Mo) and chloride (Cl⁻) were determined by ICP-OES.

Soil enzyme activities were assayed according to methods described in *Soil Enzymes and Their Research Methodology* [21]. Specifically, phenol oxidase activity was determined using the L-DOPA method, peroxidase activity by the pyrogallol colorimetric method, β-glucosidase and N-acetylglucosaminidase activities by substrate-induced methods, urease activity by the phenol-sodium colorimetric method, and protease activity by the Folin-phenol method.

### DNA extraction, illumina sequencing, and data interpretation

Total soil DNA was extracted using the DNeasy^®^ PowerSoil^®^ Kit (QIAGEN, Germany). The concentration and purity of the extracted DNA were assessed using a NanoDrop-2000 spectrophotometer (Thermo Fisher Scientific, USA), and DNA integrity was evaluated by 1% agarose gel electrophoresis. The extracted DNA served as the template for PCR amplification. The V4 region of the bacterial 16S rRNA gene was amplified using the primer pair 515 F (5′-GTGCCAGCMGCCGCGG-3′) and 806R (5′-GGACTACHVGGGTWTCTAAT-3′) [22], and the fungal ITS1 region was amplified using the primer pair 1737 F (5′-CTTGGTCATTTAGAGGAAGTAA-3′) and 2043R (5′-GCTGCGTTCTTCATCGATGC-3′) [23]. Amplicon libraries were sequenced on the Ion S5™ XL platform (Novogene Bioinformatics Technology Co., Ltd., Beijing, China).

Raw sequences were processed using Cutadapt (v1.9.1) [24] to remove low-quality reads and adapter sequences, and chimeric reads were subsequently filtered using UCHIME [25] to obtain clean reads. Operational taxonomic units (OTUs) were clustered at 97% sequence similarity using UPARSE (v7.0.1001) [26], with a representative sequence selected for each OTU. Taxonomic annotation was performed using the Mothur method [27] against the SILVA 132 database [25] with a confidence threshold of 0.8–1. Community composition was subsequently analyzed at the phylum, class, order, family, genus, and species levels. Multiple sequence alignment was conducted using MUSCLE (v3.8.31) [28].

Alpha diversity indices, including Observed species, Chao1, Shannon, and Simpson, were calculated using QIIME (v1.9.1). Differences in alpha diversity between groups were assessed using Student’s t-test or the Wilcoxon rank-sum test. Beta diversity was evaluated based on Bray–Curtis dissimilarity using permutational multivariate analysis of variance (PERMANOVA) and visualized by non-metric multidimensional scaling (NMDS). Linear discriminant analysis effect size (LEfSe) was performed to identify differentially abundant taxa (LDA score > 4.0). Relationships between environmental factors and microbial community structure were assessed using redundancy analysis (RDA) implemented in the vegan package (R v2.15.3), with significant environmental drivers identified using the envfit function (*P* < 0.05).

### Data analysis

Significant differences in soil physicochemical properties and enzyme activities between sample groups were analyzed using independent-samples *t*-tests (*P* < 0.05) in IBM SPSS Statistics 26 (IBM Corp., Armonk, NY, USA). Circular charts illustrating the relative abundance of microbial taxa at the phylum level across different samples were generated using Origin 2021 (OriginLab, Northampton, MA, USA). Bar charts displaying relative abundance at the genus level were created with GraphPad Prism 10 (GraphPad Software, San Diego, CA, USA). All figures were subsequently edited, assembled, and finalized using Adobe Illustrator 2021 (Adobe Inc., San Jose, CA, USA).

## Results

### Rhizosphere soil physicochemical properties and enzyme activities differences between healthy and diseased tobacco plants

Compared with the healthy tobacco rhizosphere (CK), the diseased tobacco rhizosphere (FB) exhibited significant alkalization (pH increased by 46.46%), accompanied by notable enrichment in soil organic matter, total nitrogen, available nitrogen, and available phosphorus, with available phosphorus showing the largest increase (112.95%). Conversely, contents of total potassium, available potassium, boron, and sulfur were significantly reduced (Table 1). Furthermore, peroxidase, urease, and protease activities in the FB rhizosphere were significantly elevated by 566.75%, 55.05%, and 35.46%, respectively, compared with CK (Table 2), indicating a close association between disease occurrence and alterations in the soil biochemical environment.

**Table 1:** Physicochemical properties of the rhizosphere soil from healthy and diseased tobacco plants

Chemical properties	Rhizosphere soil of healthy plants	Rhizosphere soil of diseased plants	*P*-value
pH	4.29 ± 0.04	6.28 ± 0.02	< 0.05
Organic Matter(g/kg)	14.71 ± 0.50	21.08 ± 0.66	< 0.05
Total Nitrogen(g/kg)	1.07 ± 0.01	1.42 ± 0.02	< 0.05
Available Nitrogen(mg/kg)	124.36 ± 1.61	211.32 ± 6.77	< 0.05
Total phosphorus(g/kg)	1.63 ± 0.22	2.16 ± 0.16	> 0.05
Available Phosphorus(mg/kg)	94.72 ± 5.78	201.71 ± 10.34	< 0.05
Total Potassium(g/kg)	22.15 ± 0.14	20.23 ± 0.41	< 0.05
Available Potassium(mg/kg)	571.19 ± 3.63	553.41 ± 2.09	< 0.05
Exchangeable Calcium(mg/kg)	1009.23 ± 2.09	2647.33 ± 488.33	> 0.05
Exchangeable Magnesium(mg/kg)	311.37 ± 17.69	673.83 ± 103.59	> 0.05
Available Copper(mg/kg)	1.13 ± 0.01	2.12 ± 0.08	< 0.05
Available Zinc(mg/kg)	1.05 ± 0.15	2.06 ± 0.08	< 0.05
Available Iron(mg/kg)	16.07 ± 0.48	16.95 ± 0.32	> 0.05
Available Manganese(mg/kg)	17.69 ± 0.68	29.47 ± 1.14	< 0.05
Available Boron(mg/kg)	1.32 ± 0.02	0.66 ± 0.09	< 0.05
Available Sulfur(mg/kg)	153.37 ± 0.90	55.22 ± 1.50	< 0.05
Available Molybdenum(mg/kg)	0.62 ± 0.04	0.60 ± 0.02	> 0.05
Chloride Ion(mg/kg)	7.64 ± 0.47	7.54 ± 0.46	> 0.05

Data in the table are presented as mean ± standard error (*n* = 3)

*P*-values were derived from independent samples t-tests in SPSS

**Table 2:** Enzyme activity characteristics in rhizosphere soil of healthy and diseased tobacco plants

Enzyme activity type	Rhizosphere soil of healthy plants	Rhizosphere soil of diseased plants	*P*-value
PO(µmol h^− 1^ g^− 1^ dry soil)	19.03 ± 8.94	23.95 ± 3.64	> 0.05
PER(µmol h^− 1^ g^− 1^ dry soil)	4.03 ± 1.79	26.87 ± 2.96	< 0.05
PNG(µmol h^− 1^ g^− 1^ dry soil)	0.51 ± 0.03	0.43 ± 0.04	> 0.05
NAG(µmol h^− 1^ g^− 1^ dry soil)	0.22 ± 0.03	0.18 ± 0.01	> 0.05
UR(µg·g^− 1^ dry soil h^− 1^)	7.63 ± 0.62	11.83 ± 1.11	< 0.05
PR(µg·g^− 1^ dry soil h^− 1^)	286.67 ± 24.09	388.33 ± 11.09	< 0.05

Data in the table are presented as mean ± standard error (*n* = 3)

*P*-values were derived from independent samples t-tests in SPSS

*PO* Phenol Oxidase, *PER* Peroxidase, *PNG* β-Glucosidase, *NAG* N-Acetyl-β-Glucosaminidase, *UR* Urease, *PR* Protease

### Diversity of rhizosphere microbial communities in diseased and healthy tobacco

**Fig. 1:**
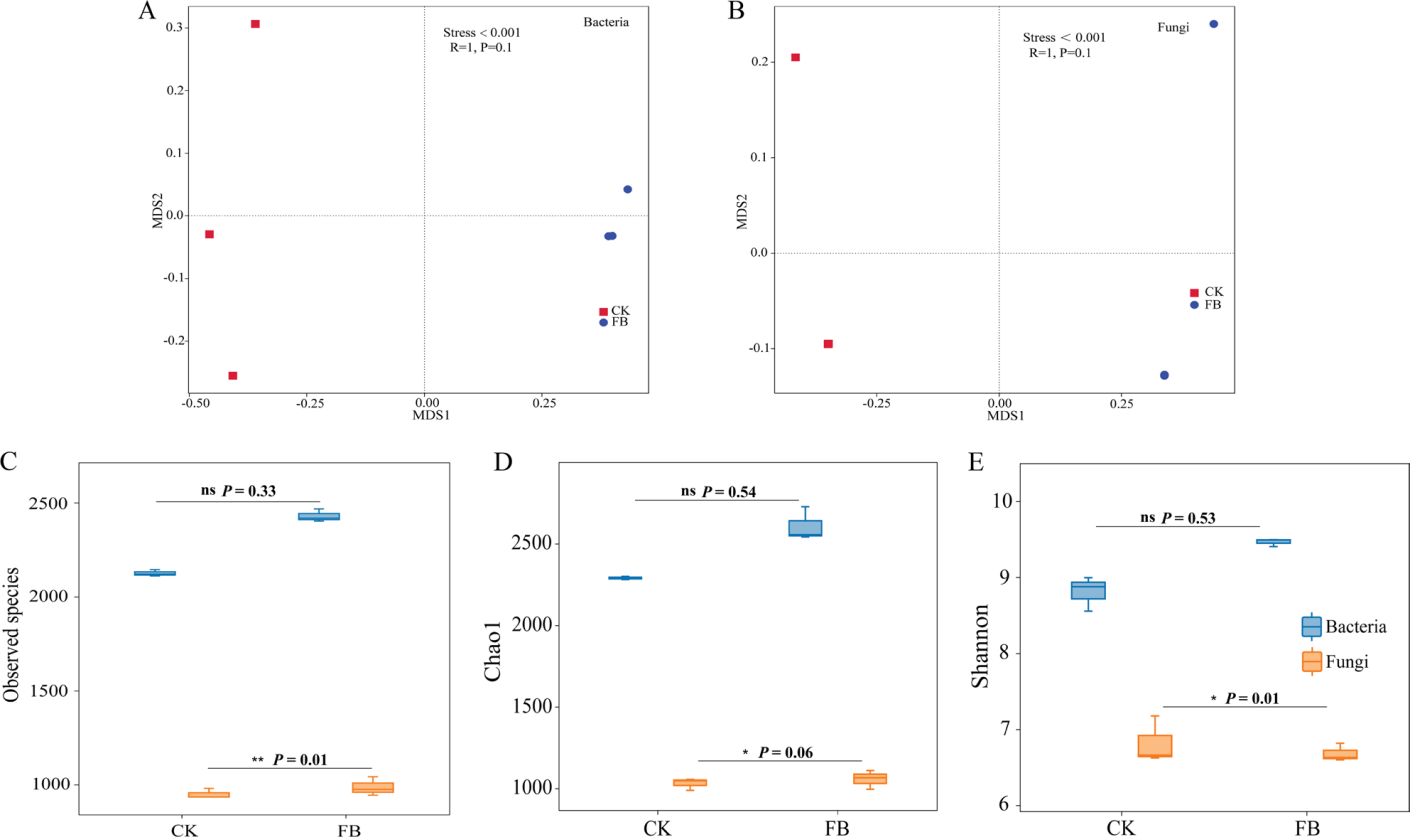
The NMDS plots illustrate the degree of dissimilarity in bacterial (**A**) and fungal (**B**) species within the rhizosphere soil of healthy (CK) and diseased (FB) tobacco plants. Significant differences in observed species (**C**), Chao1 index (**D**), and Shannon index (**E**) of bacterial and fungal communities between diseased and healthy tobacco rhizosphere soils were determined using independent samples t-test (following verification of normality and homogeneity of variance by Shapiro-Wilk and Levene’s tests). Box plots display the median (horizontal line), 25th – 75th percentiles (box), and range (whiskers), with superimposed points representing individual values (*n* = 3). * indicates *P* < 0.05, ** indicates *P* < 0.01. Mean ± standard deviation (SD) values are provided in Supplementary Table S2

Sequencing data quantity, quality, and rarefaction curves indicated sufficient depth to characterize the microbial communities (Supplementary Table 1, Supplementary Fig. 1). NMDS analysis revealed significant separation of bacterial and fungal communities between diseased (FB) and healthy (CK) rhizosphere soils along the NMDS1 axis (Fig. 1A, B), suggesting that disease status was the primary factor driving microbiome variation. Alpha diversity analysis indicated that the Observed species, Chao1, and Shannon indices for bacteria in the FB rhizosphere were significantly increased by 14.32%, 13.91%, and 7.38%, respectively, compared with CK (*P* < 0.05, independent-samples *t*-test), demonstrating that pathogen infection significantly altered bacterial community richness and diversity. In contrast, fungal community diversity exhibited no significant differences between FB and CK (*P* > 0.05) (Fig. 1C–E).

### Composition of rhizosphere soil microbial communities in diseased and healthy tobacco

**Fig. 2:**
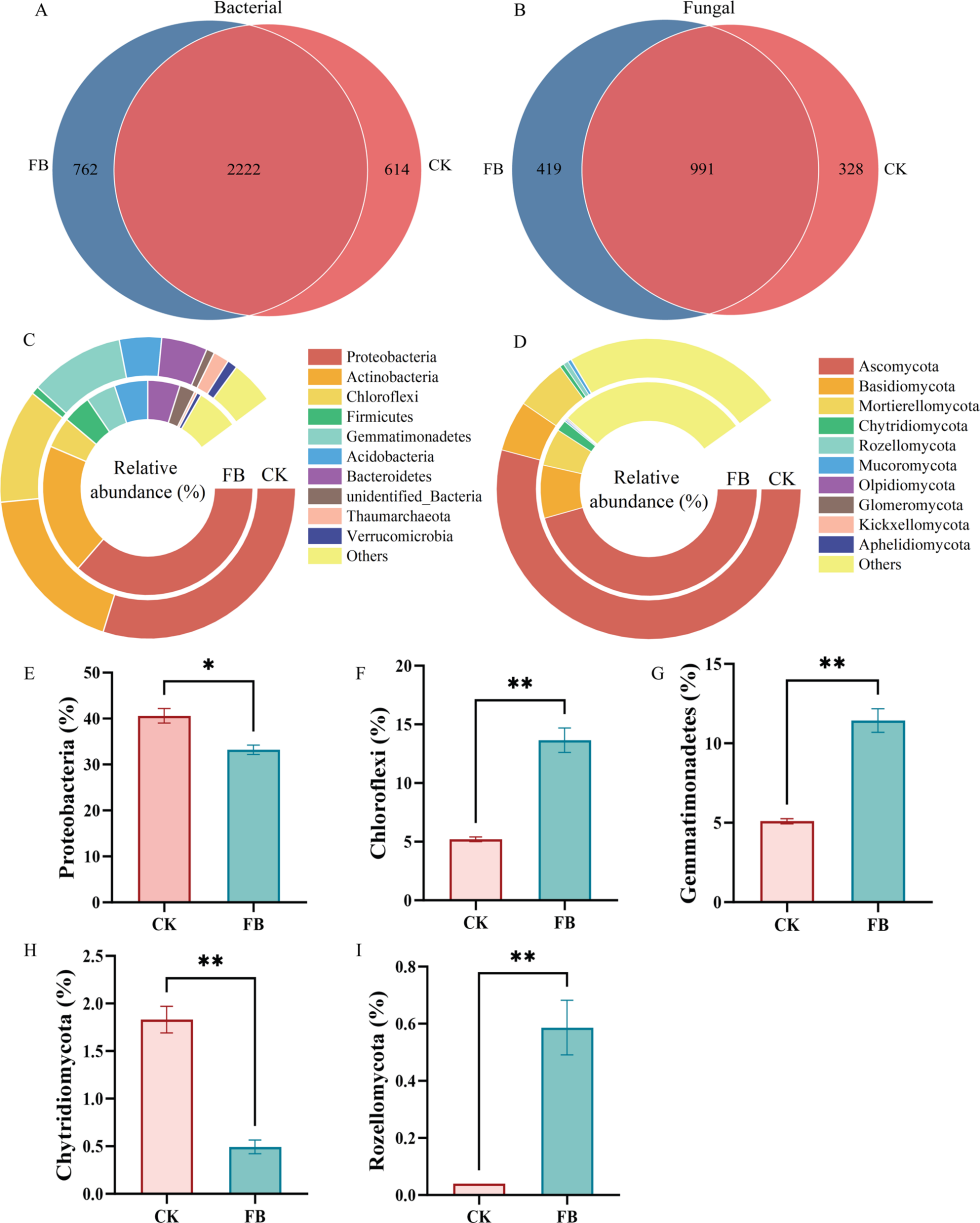
Venn diagrams show the number of identifiable bacterial (**A**) and fungal (**B**) species in the rhizosphere soil of diseased (FB) and healthy (CK) tobacco plants. Circular charts display the taxonomic information of the top 10 bacterial (**C**) and fungal (**D**) phyla in the rhizosphere soil of FB and CK. Panels **E**–**G** show the relative abundances of representative bacterial taxa in the rhizosphere soils of FB and CK. Panels **H** and **I** show the relative abundances of representative fungal taxa in the rhizosphere soils of FB and CK. Differences between groups were analyzed using independent samples *t*-test in SPSS. Significance levels are indicated as *P < 0.05 and **P < 0.001

A total of 3,598 bacterial OTUs and 1,738 fungal OTUs were obtained. Shared OTUs accounted for 61.76% and 57.02% of the total in CK and FB, respectively, whereas FB-specific OTU proportions (21.18% and 24.11%) were slightly higher than those of CK (17.07% and 18.87%) (Fig. 2A, B). Bacterial communities were dominated by Proteobacteria, Actinobacteria, and Chloroflexi, among which Proteobacteria significantly decreased by 18.20% in FB, while Chloroflexi and Gemmatimonadetes significantly increased 2-fold (*P* < 0.05) (Fig. 2C, E–G). Ascomycota and Basidiomycota were the dominant fungal phyla. Notably, Chytridiomycota significantly decreased by 72.68% in FB, whereas Rozellomycota significantly increased more than 10-fold (*P* < 0.05) (Fig. 2D, H–I).

**Fig. 3:**
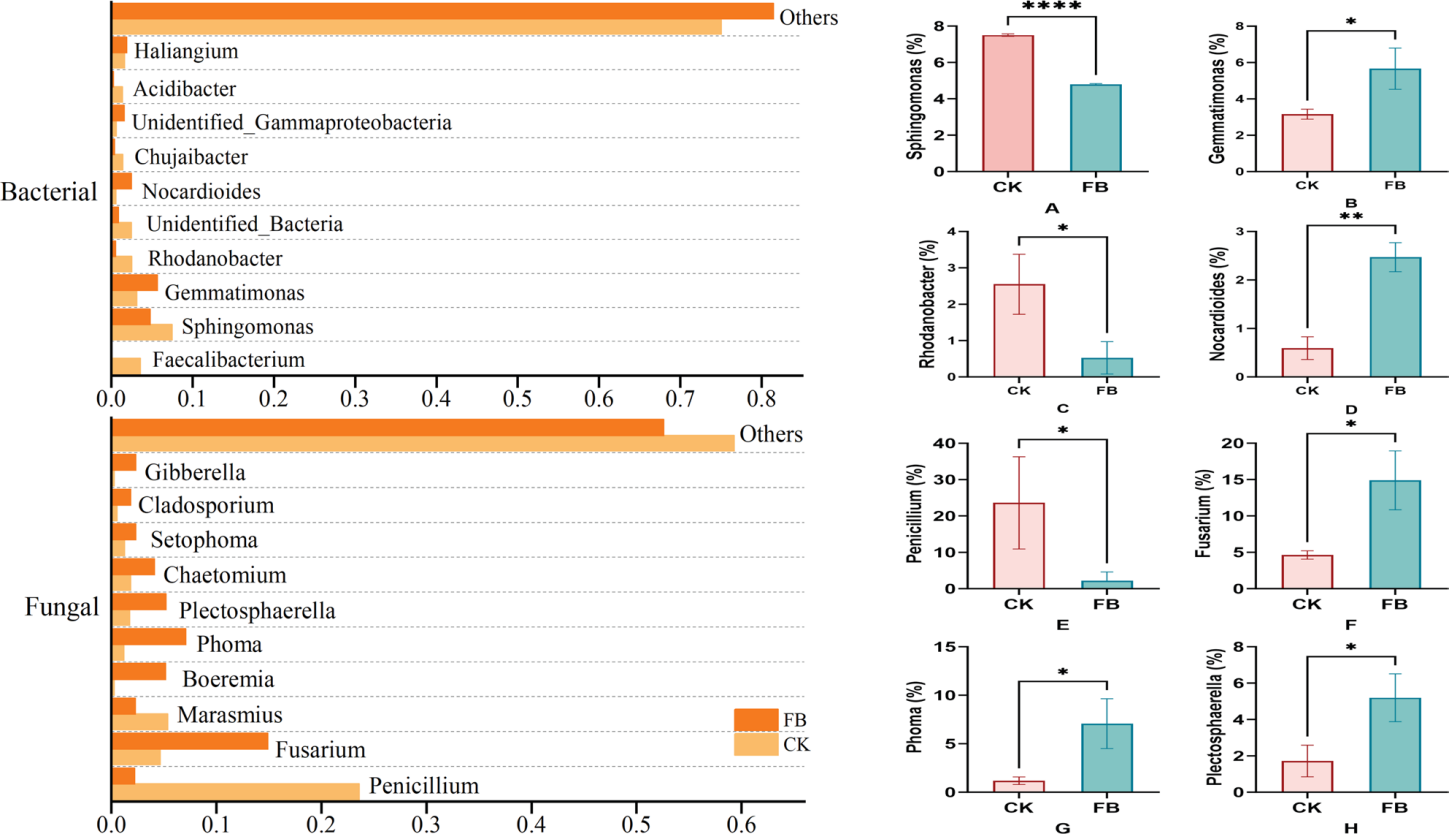
Relative abundances of representative bacterial (**A**-**D**) and fungal (**E**-**H**) taxa at the genus level in the rhizosphere soil of healthy (CK) and diseased tobacco (FB). Statistical differences were determined by independent samples *t*-test using SPSS. Significance is indicated as *P < 0.05, **P < 0.001, and ****P < 0.0001

At the genus level, dominant bacterial taxa in tobacco rhizosphere soil included *Sphingomonas*, *Rhodanobacter*, and *Gemmatimonas*, whereas dominant fungal taxa included *Penicillium*, *Fusarium*, and *Marasmius* (Fig. 3). Compared with CK, the FB rhizosphere exhibited significant decreases in beneficial bacterial genera *Sphingomonas* (36.13%) and *Rhodanobacter* (79.22%), concomitant with significant increases in *Gemmatimonas* (79.43%) and *Nocardioides* (318.64%) (*P* < 0.05) (Fig. 3A–D). Furthermore, the non-pathogenic fungus *Penicillium* significantly decreased by 90.59%, whereas the pathogenic fungi *Fusarium*, *Phoma*, and *Plectosphaerella* significantly increased by 3- to 5-fold (*P* < 0.05) (Fig. 3E–H), indicating that disease occurrence was accompanied by a directional shift in the rhizosphere microbial community from beneficial taxa toward pathogenic taxa.

### LEfSe analysis of rhizosphere microbial communities between diseased and healthy tobacco

**Fig. 4:**
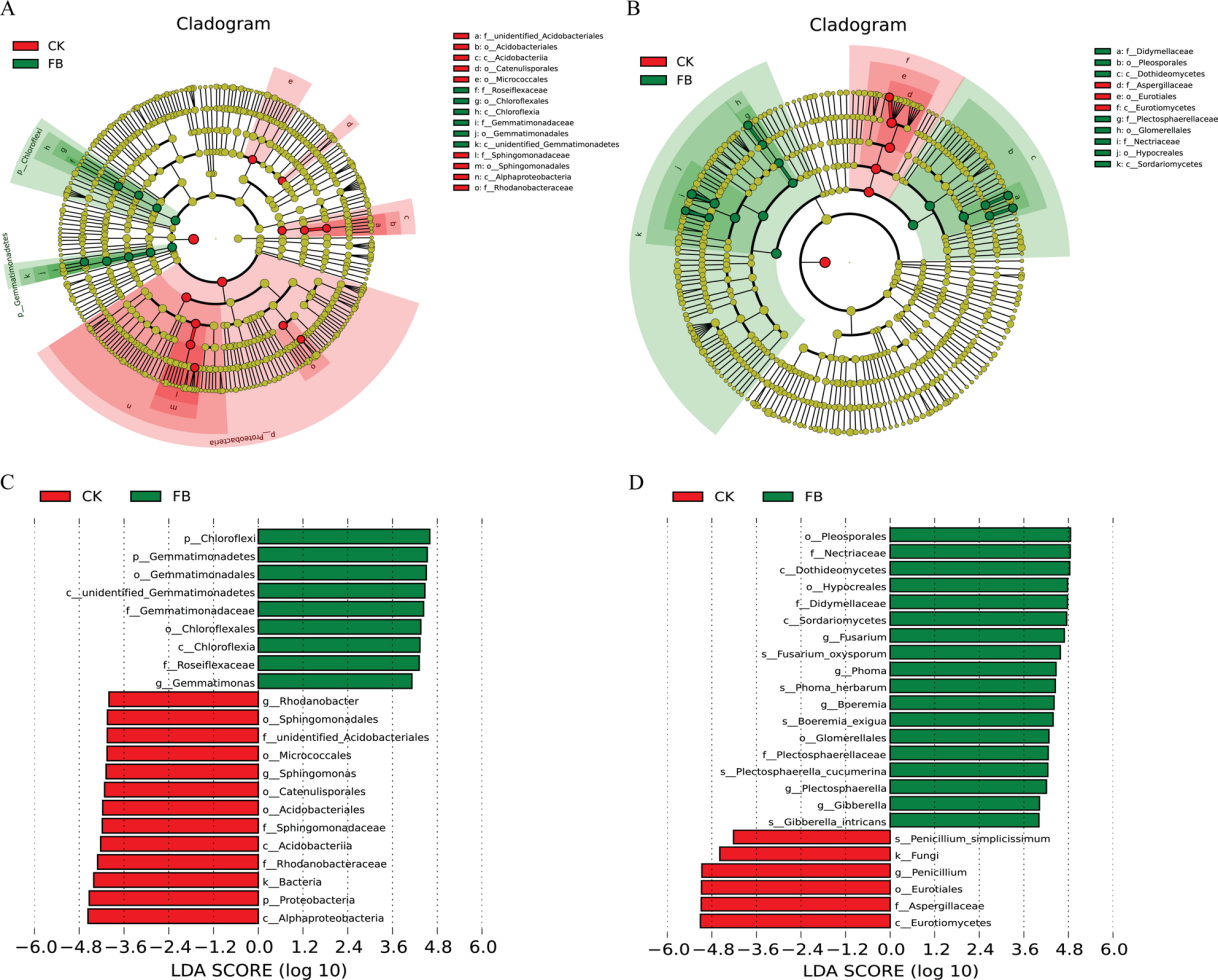
LefSe analysis of differential species. Cladograms illustrate the differential species and their relative abundances at various taxonomic levels in the rhizosphere soil samples of healthy (CK) and diseased (FB) tobacco plants for bacteria (**A**) and fungi (**B**), respectively. The radiating circles from the center outward represent the taxonomic levels from phylum to genus (or species). Each small circle at a given level represents a taxon at that level, with its diameter being proportional to its relative abundance. LDA score distribution histograms display the species with significantly differential abundances in the bacterial (**C**) and fungal (**D**) communities across different samples (species with an LDA Score > 4 are identified as statistically significant biomarkers)

LEfSe (LDA Effect Size) analysis revealed distinct microbial biomarkers between the two groups. The six differential bacterial biomarkers identified in the FB group were predominantly affiliated with *Chloroflexi* (LDA = 4.60) and *Gemmatimonadetes* (LDA = 4.53), whereas the nine bacterial biomarkers in the CK group were primarily associated with *Proteobacteria* (dominated by the class *Alphaproteobacteria*, LDA = 4.57) and *Acidobacteria*. Fungal biomarkers in both groups belonged to *Ascomycota*; however, the FB group was characterized by *Pleosporales* (LDA = 4.85), *Nectriaceae* (LDA = 4.84), and *Dothideomycetes* (LDA = 4.83), while the CK group was dominated by *Eurotiomycetes* (LDA = 5.11) and *Aspergillaceae* (LDA = 5.08) (Fig. 4). These results indicate significant differences in microbial community structure between CK and FB rhizosphere soils.

### Correlation analysis between microbial community structure and soil physicochemical properties

**Fig. 5:**
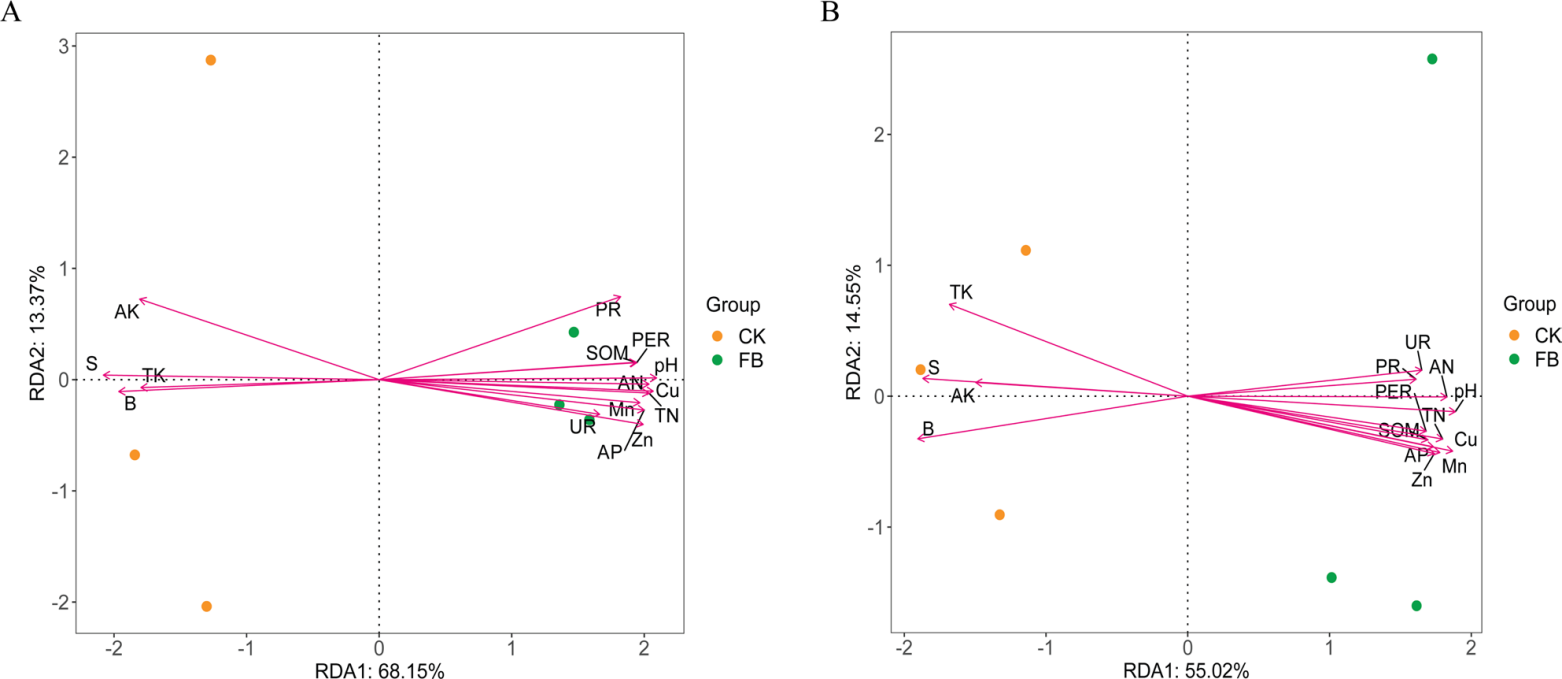
Redundancy analysis (RDA) of different soil environmental factors with bacterial (**A**) and fungal (**B**) community structures. CK, rhizosphere soil of healthy tobacco; FB, rhizosphere soil of diseased tobacco. The percentage of variation explained by each axis is indicated. Arrows represent soil physicochemical factors; the length of an arrow line indicates the influence of the corresponding soil variable on the soil microbial community structure. These factors include soil pH, soil organic matter (SOM), total nitrogen (TN), available nitrogen (AN), available phosphorus (AP), total potassium (TK), available potassium (AK), available copper (Cu), available zinc (Zn), available manganese (Mn), available boron (B), available sulfur (S), peroxidase (PER), urease (UR), and protease (PR)

Redundancy analysis (RDA) revealed significant spatial separation between CK and FB rhizosphere soils driven by environmental factors including pH, soil organic matter (SOM), nitrogen and phosphorus nutrients, and enzyme activities (Fig. 5). These environmental factors explained 81.52% of the variation in bacterial communities and 69.57% in fungal communities, with pH, total nitrogen (TN), available phosphorus (AP), and protease and urease activities identified as the key drivers distinguishing microbial community structures between the two groups.

**Fig. 6:**
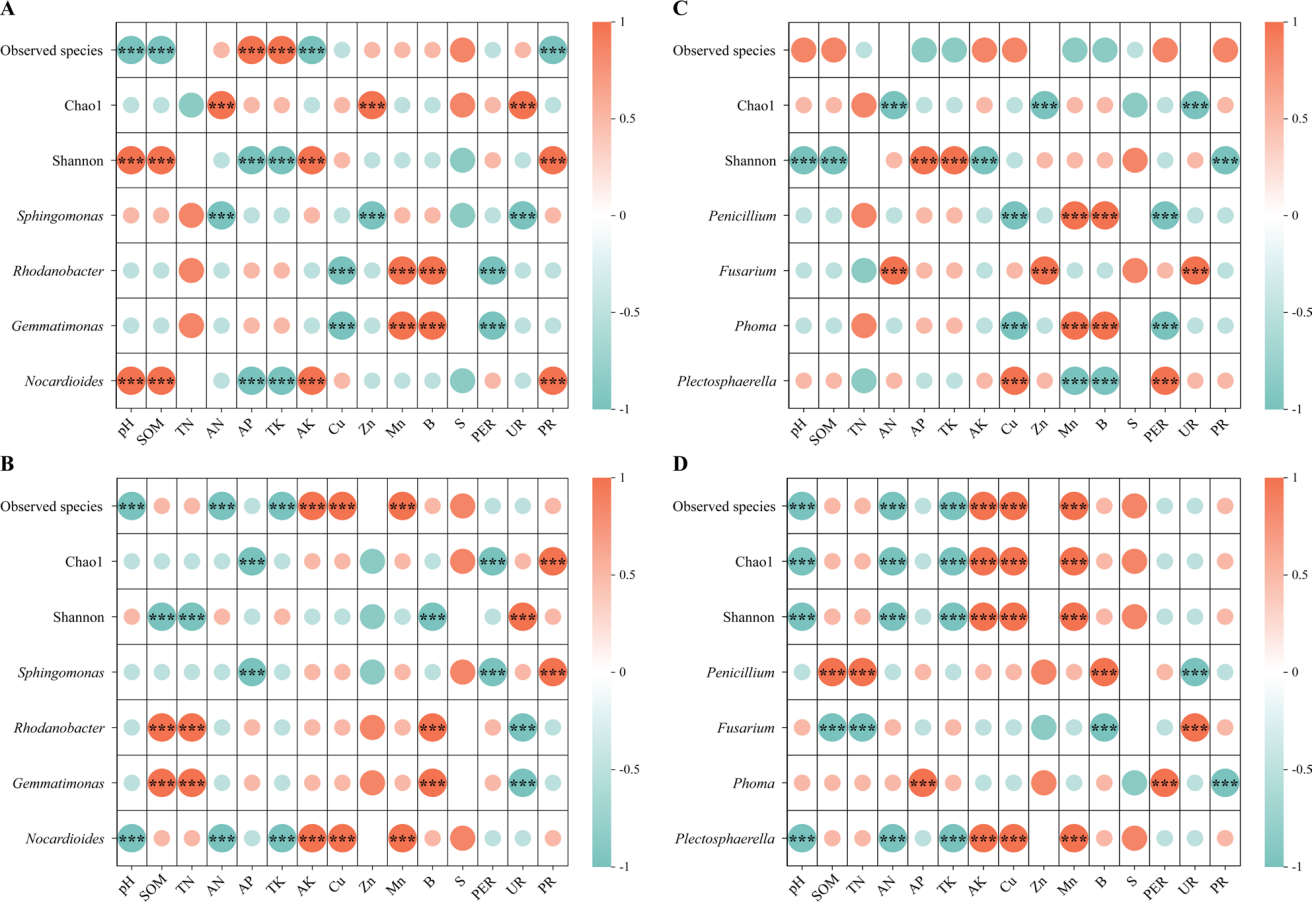
Spearman correlation analysis between the rhizosphere soil microbial community and soil physicochemical factors. **A** bacterial community in healthy tobacco rhizosphere soil; **B** bacterial community in diseased tobacco rhizosphere soil; **C** the fungal community in healthy tobacco rhizosphere soil; **D** the fungal community in diseased tobacco rhizosphere soil

Spearman correlation analysis further revealed significantly divergent association patterns between microbial diversity and environmental factors in CK and FB rhizosphere soils (Fig. 6). Specifically, bacterial diversity (Observed species, Chao1, and Shannon indices) in the FB group was negatively correlated with total potassium (TK) and available boron (B) contents, but positively correlated with peroxidase and urease activities. Fungal diversity was negatively correlated with pH, available nitrogen (AN), and TK, but positively correlated with available potassium (AK), available copper (Cu), and manganese (Mn). At the genus level, the decline of beneficial taxa (*Sphingomonas* and *Penicillium*) was closely associated with elevated soil pH, organic matter, and nitrogen nutrients, whereas the enrichment of pathogenic taxa (*Fusarium* and *Plectosphaerella*) was linked to deficiencies in TK and B, as well as altered enzyme activities. These results suggest that the imbalance between soil chemical properties and biological functions is closely associated with the deterioration of the rhizosphere microecological environment.

## Discussion

### Imbalance of rhizosphere microecosystem under mixed infection by multiple pathogens

Bacterial wilt (*Ralstonia solanacearum*), black shank (*Phytophthora parasitica*), root rot, and root-knot nematodes are soil-borne diseases frequently encountered during tobacco cultivation. These pathogens often infect the underground parts of tobacco plants individually or in mixed infections, destroying the vascular transport system and impairing the uptake of essential nutrients, consequently leading to leaf yellowing, stem blackening, and, in severe cases, whole-plant wilting and death, ultimately resulting in reduced yield and quality [29, 30]. Generally, higher alpha diversity indices indicate more complex microbial community structures and greater stability, which may reduce the risk of soil-borne pathogen infection 22, 31]. However, in the present study, bacterial alpha diversity (Observed species, Chao1, and Shannon indices) in diseased soil was significantly higher than that in healthy soil (Fig. 1C–E), contradicting the conclusion that “healthy soils harbor higher microbial diversity” reported in previous studies [10, 11, 32, 33]. We speculate that this abnormal increase resulted from the elevation of rhizosphere microbial diversity induced by pathogen invasion. Such changes typically represent a response of indigenous rhizosphere species to pathogen infection: under pathogen stress, plants usually secrete specific root exudates (sugars, amino acids, organic acids, fatty acids, and secondary metabolites) to attract and recruit microorganisms to the rhizosphere, thereby increasing local species diversity. Some of these beneficial microorganisms can protect host plants by inhibiting soil-borne pathogens or inducing systemic resistance [4, 34, 35]. However, such recruitment is not always beneficial; it may also attract additional soil-borne pathogens or harmful microorganisms to the rhizosphere, leading to microecological imbalance and exacerbating disease occurrence [36]. Similar phenomena have been reported in tomato bacterial wilt [37] and ginseng rusty root rot [14]. These observations suggest that diseased plant rhizospheres often enrich specific functional microbial groups to cope with stress, but such diversity increases do not represent enhanced ecological stability; rather, they may be a prelude to microecosystem imbalance. Notably, in this study, fungal community diversity showed no significant difference between healthy and diseased rhizosphere soils, contrasting sharply with the significant changes observed in bacterial communities. Similar results have been observed in cases of tobacco bacterial wilt [38], tomato wilt [39], and banana *Fusarium* wilt [40]. This discrepancy may arise from: (1) the greater resistance of fungi to soil environmental changes; (2) spatial niche differentiation of fungal hyphae in bulk soil [38]; and (3) differential effects of bacterial versus fungal pathogens on fungal communities 40. However, stable diversity does not imply stable function; for instance, in our study, the phylum *Chytridiomycota* significantly decreased by 72.68%, whereas the phylum *Rozellomycota* abnormally increased more than tenfold. These observations indicate that further assessment of fungal community ecological functions using functional gene prediction tools is warranted.

Furthermore, the imbalance of rhizosphere microbial community structure in diseased tobacco soils (manifested as significant reductions in *Proteobacteria* and *Chytridiomycota* and significant increases in *Chloroflexi* and *Gemmatimonadetes*) was further characterized at the genus level by an “antagonistic shift” between beneficial and pathogenic taxa: beneficial bacteria such as *Sphingomonas* (36.13%) and fungi such as *Penicillium* (90.59%) significantly decreased, while pathogenic fungi including *Fusarium*, *Phoma*, and *Plectosphaerella* were significantly enriched (3- to 5-fold). *Fusarium* is a well-known soil-borne pathogenic fungus that can induce wilting and necrosis in various plants [41]. Its parasitic or saprophytic nutritional mode likely damages plant roots and creates favorable conditions for pathogen proliferation [42, 43]. The genus *Phoma* frequently causes root and stem rot, shoot blight, and fruit necrosis in plants; specifically, *P. omnivirens* can infect tobacco stems and leaves, causing stem rot disease [44]. *Plectosphaerella* is a fungal pathogen belonging to the phylum Ascomycota and family Plectosphaerellaceae, which can cause leaf yellowing, wilting, and root necrosis in various crops [45]. Conversely, the community abundances of certain beneficial bacteria (e.g., *Sphingomonas*) and fungi (e.g., *Penicillium*) in the rhizosphere soil of diseased tobacco were significantly reduced by 36.13% and 90.59%, respectively, compared with healthy soil. Numerous studies have demonstrated that *Sphingomonas* can promote plant growth and enhance plant resistance. For instance, *Sphingobacterium tabacisoli* can induce systemic resistance in banana by accumulating defensive enzymes such as peroxidase and polyphenol oxidase, establishing a first line of defense against *Fusarium* wilt [41]. *Penicillium* participates in organic matter decomposition, promotes the cycling of carbon, nitrogen, and phosphorus, and produces antibiotics to defend against pathogen invasion 5. When attacked by pathogens, plants adjust their rhizosphere microbiome and specifically recruit beneficial microbes capable of inducing disease resistance and promoting growth to form a core microbiome, helping the soil resist pathogen invasion and thereby maximizing offspring survival probability in the soil [46]. The abrupt decline of these beneficial microorganisms suggests that they may have been outcompeted by dominant pathogens or displaced by other, more mobile, harmful microorganisms, thereby causing or aggravating pathogen invasion [42].

### Relationship between soil physicochemical properties and microbial community structure in diseased rhizosphere

In this study, rhizosphere soil pH under mixed infection by multiple pathogens was 46.39% higher than that of healthy soil, exhibiting significant alkalization. Soil organic matter, total nitrogen, available nitrogen, and available phosphorus were significantly enriched, with available phosphorus increasing by as much as 112.95%, whereas contents of total potassium, available potassium, available boron, and sulfur were significantly reduced (Table 1). Concurrently, peroxidase, urease, and protease activities were elevated by 566.75%, 55.05%, and 35.46%, respectively, relative to healthy soil (Table 2). These changes collectively reflect profound alterations in the rhizosphere habitat induced by disease. Redundancy analysis (RDA) and Spearman correlation analysis revealed that environmental factors explained 81.52% of bacterial community variation and 69.57% of fungal community variation, among which pH, total nitrogen, available phosphorus, and protease and urease activities were the key drivers distinguishing microbial community structures between the two groups (Fig. 5). At the genus level, the decline of beneficial taxa (*Sphingomonas*, *Penicillium*) was closely related to increased soil pH, organic matter, and nitrogen content, whereas the enrichment of pathogenic taxa (*Fusarium*, *Plectosphaerella*) was associated with deficiencies in total potassium and available boron, as well as changes in enzyme activities (Fig. 6). This indicates that changes in the soil chemical environment exerted strong selective pressure on rhizosphere microorganisms, with bacterial communities affected more significantly.

Previous studies have demonstrated that soil pH strongly influences microbial community composition, with neutral soils harboring higher bacterial diversity than acidic soils [47]. Moreover, soil pH can act as an environmental filter, selecting specific microbial groups and regulating community composition [48]. In our study, the increase in pH was a key selective factor for microbial community distribution, likely suppressing acidophilic beneficial taxa (e.g., *Penicillium*) and thereby creating favorable conditions for pathogen proliferation. Studies by Ahmed et al. [10] on tobacco bacterial wilt and Su et al. [49] on tomato bacterial wilt also indicated that soil pH progressively increased with disease invasion. These results suggest that the abnormal increase in soil pH is closely associated with infection by bacterial wilt and black shank pathogens. This phenomenon may arise from the release of alkaline substances such as ammonia during pathogen-induced root cell necrosis, as well as from pathogen metabolic activities (e.g., intensified ammonification) consuming large amounts of hydrogen ions, collectively elevating pH in the rhizosphere microenvironment [50]. Increased soil phosphorus availability significantly promotes the invasion of *Ralstonia* into rhizosphere microbial networks; under high-phosphorus conditions, pathogen abundance increases significantly whereas beneficial bacteria are depleted [51]. We observed similar phenomena in the rhizosphere soil of diseased tobacco plants: the substantial increase in available phosphorus (112.95%) was significantly positively correlated with the abnormal enrichment of the pathogenic fungus *Phoma*. This suggests that phosphorus enrichment may be associated with pathogen proliferation. Cao et al. [52] noted that appropriate levels of available phosphorus can reshape rhizosphere metabolic structure through plant-microbe interactions, enriching disease-suppressive microorganisms and thereby enhancing microbiome-mediated disease suppression. Based on this perspective, future experiments should be conducted in the Honghe tobacco-growing region to determine the optimal phosphorus threshold for this area. The significant reduction in total potassium, available potassium, available boron, and sulfur may reflect impaired nutrient uptake capacity of diseased plant roots and relative nutrient deficiency resulting from pathogen-plant competition.

Soil enzyme activity is significantly correlated with microbial community structure and serves as an important indicator of community functional status. In this study, the abnormal elevation of peroxidase, urease, and protease activities in diseased soil deviated from the normal range of healthy rhizosphere soil and coincided with the shift in microbial community structure from “beneficial-dominant” to “pathogen-dominant”, collectively reflecting the biochemical manifestation of functional imbalance in the rhizosphere microecosystem. The dramatic increase in peroxidase activity may further disrupt redox balance in the rhizosphere. The accumulation of reactive oxygen species (ROS) not only damages cell membrane integrity [53] but may also exert toxic effects on certain beneficial microorganisms (e.g., *Sphingomonas* and *Penicillium*), reducing their competitiveness. Meanwhile, marked increases in urease and protease activities may exert positive feedback effects on soil nitrogen mineralization and organic matter decomposition. For instance, in this study, soil organic matter, total nitrogen, and available nitrogen levels in diseased soil were significantly increased by 43.30%, 32.71%, and 69.93%, respectively, compared with healthy soil. Furthermore, redundancy analysis (RDA) indicated that soil physicochemical properties and enzyme activities explained 81.52% of bacterial community variation and 69.57% of fungal community variation, suggesting that microbial community imbalance in diseased soil is closely related to these dramatic increases in enzyme activities. However, other studies have demonstrated a significant negative correlation between tobacco root rot disease severity and protease activity (R² = 0.999, *P* < 0.05) [54]. Therefore, the correlation observed in this study requires further validation.

Despite its contributions, this study has several limitations: (1) Insufficient sample representativeness: The sampling strategy of pooling six plants into three composite replicates carries a risk of pseudoreplication, and the restriction to a single field site and single time point precludes adequate characterization of regional heterogeneity and dynamic changes across growth stages; (2) Constraints in causal inference: As an observational study, this research cannot definitively establish the direction of causality between “soil deterioration inducing disease” and “disease leading to soil alteration,” lacking validation through microcosm experiments and microbiome transplantation; (3) Methodological limitations: The current study relies solely on 16 S/ITS amplicon sequencing, without metagenomic functional analysis or absolute quantification of key taxa, making it difficult to pinpoint specific microbial sources of elevated enzyme activities; (4) Unclear mechanisms of complex infection: The relative contributions of *Ralstonia solanacearum*, *Phytophthora parasitica*, and root-knot nematodes, as well as their differential impacts on the microecosystem, remain unresolved. Future research should employ increased spatial and temporal replication, conduct controlled inoculation experiments, integrate metagenomics with metabolomics, establish single-pathogen comparative treatments, and perform microplot validation to construct a precision management system based on microecological regulation.

## Conclusion

Rhizosphere soils of tobacco plants under mixed infection by multiple pathogens in a continuously cropped field at Baishui Town, Luxi County, Honghe Hani and Yi Autonomous Prefecture, Yunnan Province, exhibited a chemical imbalance characterized by alkalization, enrichment of nitrogen and phosphorus nutrients, and depletion of potassium and boron, accompanied by markedly elevated activities of peroxidase, urease, and protease. Bacterial diversity was abnormally increased, whereas fungal communities showed no significant changes, characterized by a significant decline in beneficial taxa (*Sphingomonas* and *Penicillium*) and a significant enrichment of pathogenic taxa (*Fusarium*, *Phoma*, and *Plectosphaerella*). Furthermore, the imbalance of microbial community structure was closely associated with significant alterations in soil physicochemical properties and enzyme activities. This study preliminarily elucidated the association patterns among physicochemical factors, enzyme activities, and microbial community structure in tobacco rhizosphere soil under complex pathogen infection, providing observational evidence for understanding microecological dynamics during soil-borne disease occurrence in this region, as well as baseline data for subsequent soil amendment and microecological regulation.

## Supplementary Information

Supplementary Material 1.

## Data Availability

The raw transcriptome data have been deposited in the NCBI SRA database under project accession number PRJNA1400936 (https://www.ncbi.nlm.nih.gov/bioproject/PRJNA1400936) and PRJNA1401408 (https://www.ncbi.nlm.nih.gov/bioproject/PRJNA1401408).
