# Supplementary material for: Cross-infection by multiple soil-borne pathogens alters the physicochemical properties and microbial community structure of tobacco rhizosphere soil
Source: BMC Microbiol. 2026 May 21;26:674. doi: 10.1186/s12866-026-05173-7 (PMC13411330; doi:10.1186/s12866-026-05173-7)
Supplement: Supplementary file 1 — Supplementary Material 1. [file 12866_2026_5173_MOESM1_ESM.docx]

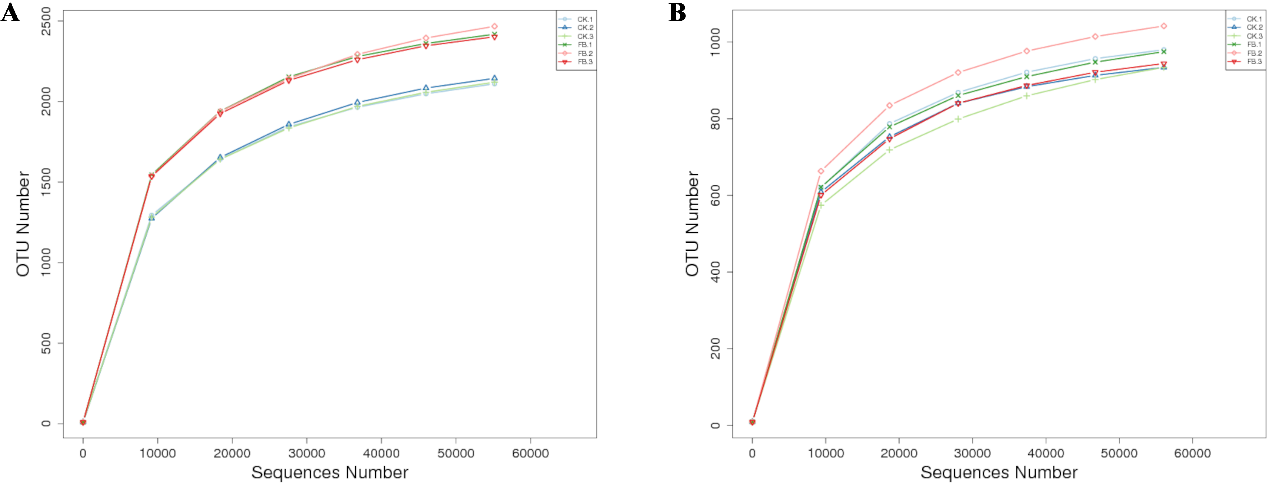


Supplementary Figure 1: Rarefaction curves of bacterial (A) and fungal (B) species based on OTUs obtained from sequencing of rhizosphere soil.

Supplementary Table 1: Sequencing quality control statistics for bacteria and fungi in rhizosphere soil of healthy and diseased tobacco.

| **Type** | **Sample name** | **Raw reads** | **Clean reads** | **Base (nt)** | **AvgLen (nt)** | **Q20 (%)** | **GC (%)** | **Effective (%)** |
| --- | --- | --- | --- | --- | --- | --- | --- | --- |
| **Bacterial** | CK.1 | 82, 977 | 80, 111 | 20, 279, 598 | 253 | 84.25 | 55.66 | 96.55 |
|  | CK.2 | 82, 417 | 80, 208 | 20, 276, 734 | 252 | 86.2 | 55.28 | 97.32 |
|  | CK.3 | 82, 323 | 80, 235 | 20, 298, 536 | 252 | 89.04 | 55.69 | 97.46 |
|  | FB.1 | 82, 745 | 80, 332 | 20, 312, 787 | 252 | 88.38 | 56.91 | 97.08 |
|  | FB.2 | 65, 434 | 64, 214 | 16, 230, 362 | 252 | 85.2 | 57.08 | 98.14 |
|  | FB.3 | 82, 040 | 80, 121 | 20, 268, 176 | 252 | 87.47 | 57.37 | 97.66 |
| **Fungal** | CK.1 | 82, 045 | 80, 121 | 19, 932, 804 | 248 | 81.45 | 50.31 | 97.65 |
|  | CK.2 | 81, 961 | 80, 264 | 19, 385, 644 | 241 | 72.65 | 52.41 | 97.93 |
|  | CK.3 | 81, 982 | 80, 217 | 18, 994, 997 | 236 | 71.88 | 52.96 | 97.85 |
|  | FB.1 | 82, 093 | 80, 101 | 17, 909, 855 | 223 | 83.17 | 47.95 | 97.57 |
|  | FB.2 | 82, 563 | 80, 242 | 18, 012, 974 | 224 | 81.1 | 48.15 | 97.19 |
|  | FB.3 | 82, 173 | 80, 030 | 17, 880, 391 | 223 | 79.86 | 46.72 | 97.39 |

Raw reads: sequences from which low-quality bases have been removed. Clean reads: sequences retained after chimera filtering and used for downstream analyses. Base: the total number of bases in the final Clean reads. AvgLen: the average length of the Clean reads. Q20: the percentage of bases in the Clean reads with a quality score ≥ 20 (corresponding to a sequencing error rate < 1 %). GC (%): the GC content of the Clean reads. Effective (%): the ratio of Clean-read count to Raw-read count, expressed as a percentage.

Supplementary Table 2: Comparison of alpha diversity indices between healthy (CK) and diseased (FB) tobacco rhizosphere soils for bacterial and fungal communities.

| **Type** | **Sample** | **Observed species** | **Chao1** | **Shannon** |
| --- | --- | --- | --- | --- |
| **Bacterial** | CK | 2124.67±17.47 | 2289.19±9.23 | 8.81±0.23 |
|  | FB | 2429.00±33.87 | 2607.65±103.04 | 9.46±0.05 |
|  | *P*-value | 0.01 | 0.06 | 0.03 |
| **Fungal** | CK | 949.33±26.56 | 1029.37±36.08 | 6.82±0.31 |
|  | FB | 987.00±50.09 | 1055.91±57.73 | 6.68±0.12 |
|  | *P*-value | 0.33 | 0.54 | 0.53 |
